# Case Report: Fibrosis appearing as disease progression on PET/CT following R-CHOP in newly diagnosed DLBCL

**DOI:** 10.3389/fonc.2026.1845484

**Published:** 2026-06-26

**Authors:** Erin A. Dean, Julia Tran, June Herman, Nicole Foley, Xiaohui Zhao

**Affiliations:** 1 Hematopoietic Stem Cell Transplantation and Cellular Therapy Program, Division of Hematology/Oncology, Department of Medicine, University of California, Irvine, Irvine, CA, United States; 2 Department of Radiological Sciences, University of California, Irvine, Irvine, CA, United States; 3 Malignant Hematology, Division of Hematology/Oncology, Department of Medicine, University of California, Irvine, Irvine, CA, United States; 4 Department of Pathology, University of California Irvine, Irvine, CA, United States

**Keywords:** case report, ctDNA, DLBCL, fibrosis, R-CHOP

## Abstract

Diffuse large B-cell lymphoma (DLBCL) is a frequently encountered aggressive lymphoma in clinical practice. The immunochemotherapy regimen consisting of rituximab/cyclophosphamide/doxorubicin/vincristine/prednisone (R-CHOP) is a standard first-line therapy that results in complete remission in the majority of patients. Here, we summarize the case of a patient with newly diagnosed DLBCL who, after completing a full 6-cycle course of R-CHOP, exhibited increasing uptake in her original retroperitoneal mass on her end-of-therapy and 3-month post-therapy 18F-fluorodeoxyglucose (FDG)-positron emission tomography/computed tomography (PET/CT) scans. Evaluation with further imaging studies, tissue biopsies, and minimally invasive measurable residual disease tests ruled out primary refractory lymphoma. The FDG-avidity of the lesion was attributed to the presence of fibrosis, as it resolved on subsequent PET/CT scans without further therapeutic interventions. This case demonstrates how fibrosis may mimic disease progression on PET/CT after a cytotoxic regimen in DLBCL and should be considered in the differential diagnosis for patients with positive PET/CT scans to potentially spare them from receiving next-line therapy.

## Introduction

Diffuse large B-cell lymphoma (DLBCL) is the most common type of aggressive B-cell lymphoma in the Western world (1). For over two decades, the standard-of-care (SOC) treatment for newly diagnosed DLBCL has been rituximab/cyclophosphamide/doxorubicin/vincristine/prednisone (R-CHOP), which achieves a 60%–70% complete response rate (2). Anti-CD19 chimeric antigen receptor (CAR) T-cell therapy has recently become the SOC for primary refractory or early relapsed DLBCL (3). Determining therapy response prior to initiating second-line therapy is crucial for sparing patients from unnecessary treatment associated with potential adverse effects. Here, we present the case of a patient referred to our academic center for CAR T-cell therapy evaluation due to suspected primary refractory DLBCL post-R-CHOP, but who was instead found to have therapy-related changes.

## Case description

A woman in her 60s, with a medical history significant for hypertension, atrial fibrillation, right lower extremity deep venous thrombosis, hiatal hernia complicated by gastroesophageal reflux disease, and surgical history of cholecystectomy and C-section, was seen at our cancer center for further evaluation and management of presumed primary refractory DLBCL. Regarding her lymphoma, the patient initially presented in August 2024 with right-sided back pain but no constitutional symptoms. A computed tomography (CT) scan of the abdomen/pelvis revealed multiple retroperitoneal masses, measuring up to 9 cm, causing right-sided hydronephrosis, which necessitated nephrostomy tube placement. Chest CT and a bone marrow biopsy showed no evidence of disease. The patient underwent a CT-guided retroperitoneal mass biopsy, which was consistent with DLBCL, activated B-cell (ABC) subtype, with a Ki-67 of 80%–90%; FISH was not performed (**Figure 1A**).

**Figure 1:**
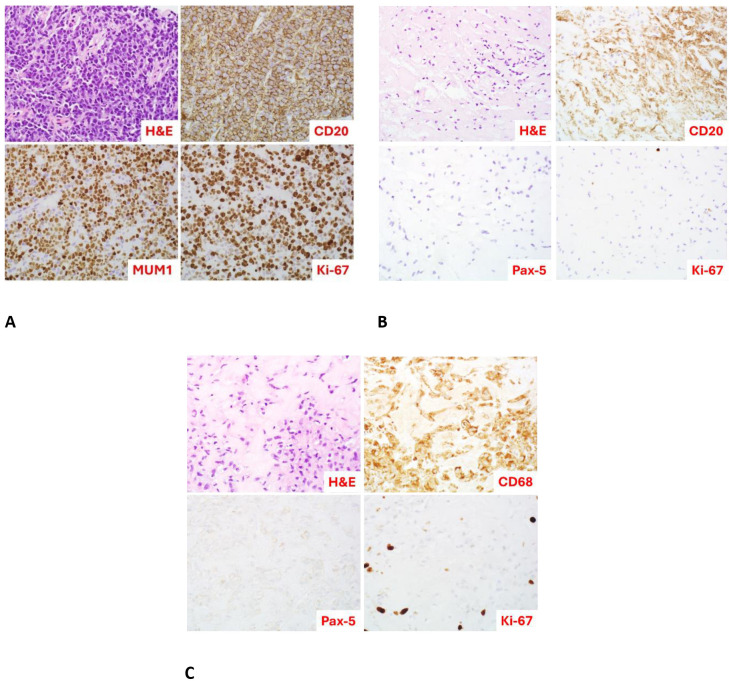
Biopsy results throughout the patient’s treatment course. The hematoxylin and eosin (H&E) stained core biopsy of the para-aortic lymph node. **(A)** At diagnosis, it reveals sheets of large lymphocytes positive for CD20 and MUM1 with a high proliferation index by ki-67 (90%), consistent with diffuse large B-cell lymphoma (DLBCL), non-germinal center B-cell (non-GCB) or activated B-cell (ABC) subtype. **(B)** At end of chemotherapy, it reveals fragments of fibrous tissue with scattered T cells and histiocytes with no residual B-cell lymphoma. CD20 stains tissue matrix rather than cells. PAX5 is negative. Ki-67 showed a low proliferation index. **(C)** Four months post-chemotherapy, it reveals fragments of fibrous tissue with scattered T cells and histiocytes (by CD68 stain) with no residual B-cell lymphoma. PAX5 is negative. Ki-67 showed a low proliferation index.

She was diagnosed with Stage II DLBCL, ABC subtype, and began first-line therapy with R-CHOP administered by her local oncologist. An interim 18F-fluorodeoxyglucose (FDG)-positron emission tomography (PET)/CT scan, performed after four cycles of chemotherapy, indicated a partial response. Treatment was interrupted for 2 months due to a port infection requiring hospitalization. A repeat PET/CT scan, conducted after the infection resolved, showed stable disease with a Deauville score (DS) of 4, unchanged from the interim scan. The patient then received two additional cycles of R-CHOP to complete the chemotherapy course.

Her end-of-therapy PET/CT scan, performed 4 weeks later in April 2025, revealed a persistent hypermetabolic soft tissue mass in the right lower para-aortic/proximal common iliac region, measuring 4.9 cm × 2.6 cm. This mass showed a slight increase in FDG activity, with a standardized uptake value (SUV) max of 6.2, up from 4.8 previously, and a DS of 4. Differential diagnoses included refractory DLBCL, post-treatment inflammation, fibrosis, and infection. A CT-guided biopsy of the right periaortic lymph node was performed to clarify the response. It was negative for malignancy but contained fragments of fibrous tissue with scattered T cells and histiocytes (**Figure 1B**). The patient was subsequently placed under close surveillance.

A restaging PET/CT scan in July 2025 showed an interval increase in FDG activity within the right lower para-aortic soft tissue mass compared to the previous two scans. The SUVmax increased to 10.1 (re-measured at our center as 7.0) from 6.1 previously, with the mass measuring approximately 3.5 cm × 2.4 cm. Additionally, two small foci of increased FDG activity were observed in the lower para-aortic/adrenal region and along the left lower para-aortic region, with a DS of 5 (**Figure 2A**). At this time, the patient’s laboratory results were normal, including her complete metabolic panel (CMP), complete blood cell counts with differential (CBC with diff), and lactate dehydrogenase (LDH) at 119 U/L (normal range: 96–199 U/L).

**Figure 2:**
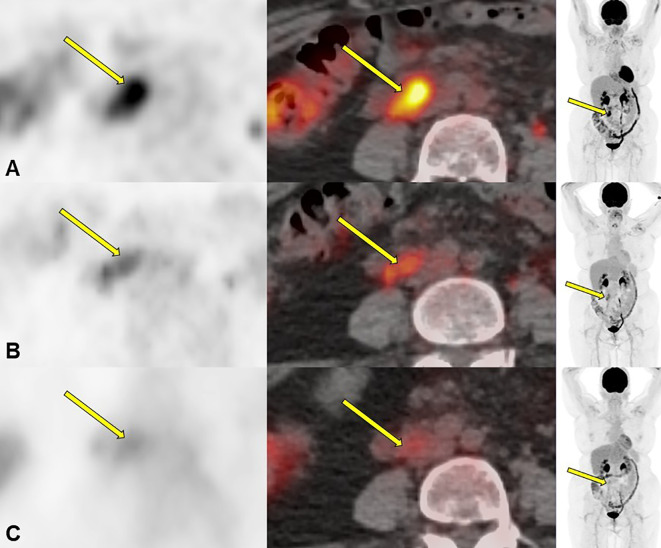
Serial follow-up FDG-PET/CT images after the end-of-therapy restaging. Axial FDG-PET (left), fused PET/CT (middle), and MIP (right) at the pericaval retroperitoneal soft tissue lesion (arrows) in **(A)** July 2025, SUVmax of 6.7 (hepatic SUVmean 1.6); **(B)** October 2025, SUVmax of 4.0 (hepatic SUVmean 1.6), and **(C)** December 2025, SUVmax of 2.3 (hepatic SUVmean 1.7), demonstrating progressive decline in FDG avidity without additional systemic therapy.

Given the concern for disease progression on imaging, the patient underwent another image-guided right retroperitoneal lymph node biopsy. Pathology results again showed no malignancy but revealed fibrous tissue with scattered T cells and histiocytes (**Figure 1C**). She was referred to our academic center for evaluation for potential CAR T-cell therapy. During her consultation, the patient reported a stable sensation in her back, present since diagnosis, but denied any B symptoms. Her nephrostomy tube had been removed shortly after chemotherapy initiation.

Further workup was performed to determine her disease status. A magnetic resonance imaging (MRI) of the abdomen/pelvis with and without contrast in September 2025 revealed a 2.4 cm × 1.4 cm confluent nodal mass in the right lower retroperitoneum and right common iliac chain, concerning for nodal manifestation of the disease (**Figure 3**). However, a repeat PET/CT scan in October 2025 showed a pericaval retroperitoneal lesion with an interval decrease in size and activity from July 2025, with an SUVmax of 4.0 (previously 6.7) and a size of 2.4 cm. This was interpreted as an area of focal retroperitoneal fibrosis with decreasing inflammation, although active disease remained in the differential diagnosis (DS 3) (**Figure 2B**). Peripheral blood circulating tumor DNA (ctDNA) by next-generation sequencing was undetectable. Based on these results, continued surveillance was recommended.

**Figure 3:**
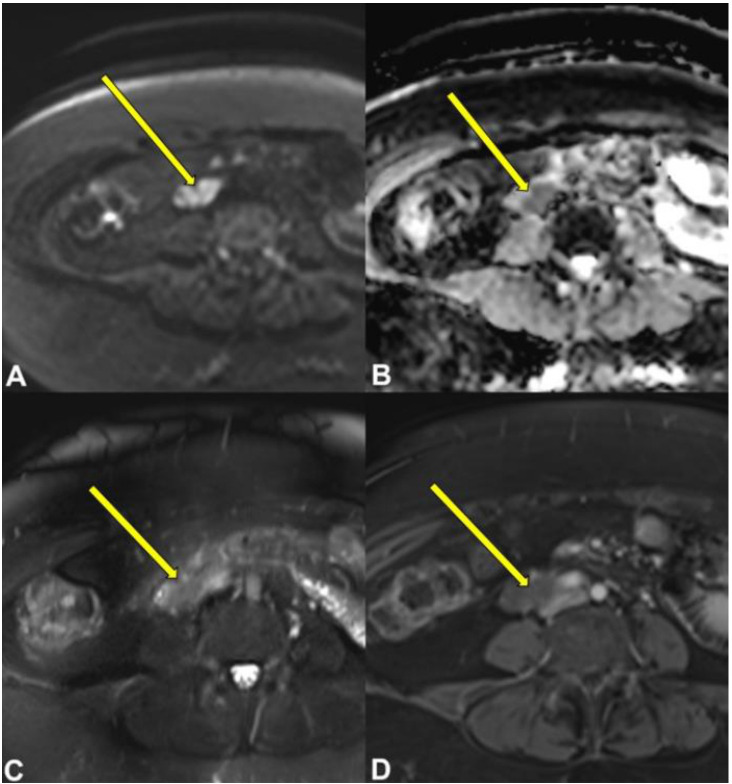
MRI of the abdomen/pelvis 5 months post first-line therapy for DLBCL. Axial **(A)** DWI, **(B)** ADC, **(C)** T2-weighted, and **(D)** T1-weighted post-contrast images in September 2025 show a 2.4 cm × 1.4 cm right paracaval retroperitoneal lymph node (arrows) with restricted diffusion, T2 mild hyperintensity, and hypoenhancement, concerning for disease.

On reevaluation 3 months later, she remained asymptomatic. Another restaging PET/CT scan demonstrated no evidence of FDG-avid recurrent lymphoproliferative disease (DS 3) (**Figure 2C**). LDH was within normal limits at 178 U/L, and ctDNA was again undetectable. CMP and CBC with differential were unremarkable.

At the time of this report, the patient remains in complete first remission.

## Discussion

This case demonstrates the occurrence of fibrosis leading to apparent disease progression on PET/CT scans following six cycles of R-CHOP chemotherapy in newly diagnosed DLBCL. Therapy-related changes are an unexpected and rare cause of persistently positive and worsening PET/CT scans with this commonly used chemotherapy regimen.

In addition to clinical assessment, FDG-PET/CT scans are currently the standard imaging modality for evaluating treatment response in patients with non-Hodgkin lymphoma, with nodal and extranodal FDG-avidity likely indicating lymphoma involvement (4). A Deauville score of 4 is assigned to a PET/CT scan if a lesion or lesions show moderately increased FDG uptake compared to the liver (baseline), and a score of 5 if they show markedly increased FDG uptake (4). When the Deauville score is 4 or 5 and FDG uptake is increased compared to a prior scan, or a new lesion is present, the response is marked as consistent with disease progression (5). Our patient’s end-of-therapy PET/CT scan and first follow-up scan 3 months post-treatment showed a continuous increase in FDG uptake and were assigned Deauville scores of 4 and 5, respectively. Based on the Lugano classification, her serial radiological studies were suggestive of progressive metabolic disease, which in her case would have implied a diagnosis of primary refractory lymphoma.

However, comparing PET scans from different institutions was challenging due to variations in acquisition and reconstruction protocols. Only scans performed at our institution followed a consistent protocol, allowing for a valid intra-institution trend assessment. Additionally, the end-of-therapy PET scan was conducted at 4 weeks, rather than the ideal 6 weeks post-immunochemotherapy, potentially increasing false-positive findings. Given the patient’s negative end-of-therapy biopsy, a CT scan in 6 months, instead of a repeat PET scan in 3 months, would have aligned with guidelines, preventing further false-positive imaging and repeat biopsies (3). While both lymph node biopsies were CT-guided, only the second was explicitly confirmed by Interventional Radiology to correspond to the FDG-avid lymph node on prior PET/CT, ensuring better targeting. These imaging limitations underscore the need for caution against overreliance on PET scan findings for determination of treatment response and differential diagnosis.

Abnormal imaging findings necessitated further investigation using SOC tissue biopsies and established and novel biomarkers to clarify the response. Repeat tissue biopsies, the gold standard for diagnosis, performed at the end of therapy and 3 months later, revealed fibrous tissue without residual lymphoma. The non-specific peripheral lymphoma marker, LDH, also remained within normal limits on follow-up. Although the patient’s peripheral ctDNA was not obtained pre-treatment or at the end of therapy, it was undetectable at 5 and 8 months post-therapy, coinciding with a decrease in FDG uptake and the lesion’s appearance becoming consistent with fibrosis.

ctDNA has emerged as a novel, minimally invasive biomarker for assessing peripheral measurable residual disease (MRD) in LBCL. Recent large studies support the prognostic value of ctDNA at the end of first-line therapy in LBCL. For instance, in one study of 137 patients with newly diagnosed LBCL receiving 4–6 cycles of anthracycline-based immunochemotherapy, ctDNA became undetectable in 78% of patients at the end of therapy. It demonstrated a better prognostic value compared to PET scans at the same time point, with a hazard ratio (HR) of 28.3 for detectable ctDNA versus 3.6 for positive PET (6).

As part of the DIRECT prospective, multicenter study, an analysis of end-of-therapy ctDNA from 155 patients undergoing first-line therapy for LBCL showed that the 2-year time to tumor progression was significantly better for those with undetectable MRD (95%) compared to detectable MRD (42%). Furthermore, ctDNA outperformed radiologic assessments in terms of prognosis, with an HR of 16.9 versus 6.9, respectively (7).

A real-world prospective cohort, HOVON-902, included patients undergoing first-line therapy for LBCL with either R-CHOP or dose-adjusted rituximab/etoposide/prednisone/vincristine/cyclophosphamide/doxorubicin (R-EPOCH). Analysis of end-of-therapy ctDNA in 134 patients demonstrated a 3-year progression-free survival of 17% with MRD positivity versus 85% with MRD negativity (8). The study results validated ctDNA as a prognostic marker, showing a significantly improved positive predictive value for 2-year progression-free survival (68%) compared to a positive PET/CT scan (56%) (8).

In the future, ctDNA might be used not only for prognostication but also for guiding treatment escalation and de-escalation decisions. The Alpha 3 trial is an ongoing pivotal phase 2 study investigating this concept by evaluating the value of the allogeneic CAR T-cell therapy, cemacabtagene ansegedleucel, as consolidation in LBCL patients with detectable end-of-first-line-therapy ctDNA as their only evidence of residual disease (9). All these studies detect MRD by first identifying somatic phased variants in pretreatment tumor or plasma and then tracking them in plasma samples (6). This method, still under investigation, allows for more accurate MRD detection compared to methods identifying only single-nucleotide variants, ensuring detection at the parts-per-million range from a blood sample (6).

Our patient’s ctDNA was determined via a commercially available assay that tracks B-cell receptor rearrangements. This different method has also been shown to yield a prognostic MRD test in LBCL patients post-first-line therapy (10, 11). Given that concurrent ctDNA may be undetectable in some patients despite confirmed active tumor (10), interpretation of her ctDNA results at 5 and 8 months post-therapy was limited by the lack of testing at the time of diagnosis. Her end-of-therapy ctDNA was also not checked, which would have allowed for better prognostication.

In this case, the serial post-treatment biopsies showing fibrous tissue, undetectable peripheral ctDNA tests, and resolution of FDG avidity on PET/CT of the already treated tumor without further systemic or localized interventions collectively supported a diagnosis of therapy-related fibrosis. Worsened imaging findings are not always due to tumor progression; they can also result from inflammation, infection, fibrosis, or pseudoprogression. Therefore, performing the indicated workup for a full evaluation is crucial.

Pseudoprogression is an imaging finding defined by the initial worsening of an FDG-avid lesion(s) or the appearance of a new FDG-avid lesion, followed by improvement on subsequent imaging without therapy (12). It differs from tumor flare, which also involves clinical symptoms (12). Pseudoprogression has been observed in DLBCL with various immunotherapies, such as the antibody-drug conjugate polatuzumab vedotin, the bispecific T-cell engager therapy epcoritamab, and CAR T-cell therapy. It results from the infiltration of immune cells into the tumor, causing edema, necrosis, and hemorrhage (13, 14). The R-CHOP regimen, however, is unlikely to cause pseudoprogression given its mechanism of action is primarily through cytotoxicity. The patient’s post-therapy tissue biopsies included only scattered T cells and histiocytes.

While fibrotic changes have not been observed on PET/CT due to R-CHOP, they have been reported in solid tumors. For example, in a patient with peritoneal carcinomatosis due to ovarian cancer, a PET/CT scan became positive in new areas post-treatment despite the resolution of the original lesions (15). The uptake on a subsequent PET scan subsided, and tissue biopsy showed fibrosis (15). On PET/CT, idiopathic retroperitoneal fibrosis and retroperitoneal fibrosis due to secondary causes like malignancy can mimic lymphoma. These are favored when the lesion appears in the periaortic area, is non-bulky, and demonstrates enhancement (16). Attempts to distinguish idiopathic retroperitoneal fibrosis from lymphoma and retroperitoneal malignancy on PET have led to the development of a logistic regression model. This model incorporates the location, number, and SUVmax of suspected lymph nodes, achieving an area under the curve of 0.987, a sensitivity of 90.5%, and a specificity of 98.6% (17). Further studies are needed to elucidate the difference between these entities on PET/CT.

In conclusion, this case illustrates a rare occurrence of therapy-related fibrosis leading to apparent disease progression on PET/CT post R-CHOP chemotherapy in newly diagnosed DLBCL. Evaluation by serial imaging, repeat tissue biopsies, and continuous peripheral MRD monitoring ultimately determined the patient’s response as a complete first remission, eliminating the need for further therapy. Given the prevalence of DLBCL and the frequent use of R-CHOP as a first-line therapy, accurate determination of disease status at the end of therapy, using both established and new testing methods, is crucial. This helps avoid the potential pitfalls of non-specific PET/CT imaging and prevents the unnecessary initiation of a subsequent line of therapy.

## Data Availability

The original contributions presented in the study are included in the article/supplementary material. Further inquiries can be directed to the corresponding author.
